# Gut-Brain Axis: Possible Role of Gut Microbiota in Perioperative Neurocognitive Disorders

**DOI:** 10.3389/fnagi.2021.745774

**Published:** 2021-12-22

**Authors:** Xiao-qing Wang, He Li, Xiang-nan Li, Cong-hu Yuan, Hang Zhao

**Affiliations:** ^1^Department of Anesthesiology, School of Medicine, Affiliated Yancheng Hospital, Southeast University, Yancheng, China; ^2^Department of Anesthesiology, Affiliated Shuguang Hospital, Shanghai University of Traditional Chinese Medicine, Shanghai, China

**Keywords:** gut-brain axis, perioperative neurocognitive disorders, postoperative cognitive dysfunction, gut microbiota, cognition

## Abstract

Aging is becoming a severe social phenomenon globally, and the improvements in health care and increased health awareness among the elderly have led to a dramatic increase in the number of surgical procedures. Because of the degenerative changes in the brain structure and function in the elderly, the incidence of perioperative neurocognitive disorders (PND) is much higher in elderly patients than in young people following anesthesia/surgery. PND is attracting more and more attention, though the exact mechanisms remain unknown. A growing body of evidence has shown that the gut microbiota is likely involved. Recent studies have indicated that the gut microbiota may affect postoperative cognitive function via the gut-brain axis. Nonetheless, understanding of the mechanistic associations between the gut microbiota and the brain during PND progression remains very limited. In this review, we begin by providing an overview of the latest progress concerning the gut-brain axis and PND, and then we summarize the influence of perioperative factors on the gut microbiota. Next, we review the literature on the relationship between gut microbiota and PND and discuss how gut microbiota affects cognitive function during the perioperative period. Finally, we explore effective early interventions for PND to provide new ideas for related clinical research.

## Introduction

Perioperative neurocognitive disorders (PND), mainly encompassing acute postoperative delirium (POD) and longer-lasting postoperative cognitive dysfunction, are common postoperative complications in elderly patients. They are characterized by decreased cognitive function, and they can involve psychosis, anxiety, personality changes, and memory disorders (Evered et al., 2018). PND can occur from days to months after surgery, and the duration varies. The incidence of PND varies from 41–75% at 7 days to 18–45% at 3 months postoperatively (Austin et al., 2019). PND can cause poor functional recovery, prolonged hospitalization, and increased postoperative morbidity and mortality. These effects reduce patients’ quality of life and pose a heavy economic burden on the patients, their families, and wilder society (Steinmetz et al., 2009). Therefore, it is urgent to develop effective strategies for the prevention and treatment of PND.

In recent years, mounting evidence has highlighted a prominent role for the gut microbiota in the pathophysiology of many symptoms and diseases, including Alzheimer’s disease (AD), Parkinson’s disease (PD), type 2 diabetes, and obesity (Acharya et al., 2013; Wu et al., 2020; Zhu S. et al., 2020). The gut microbiota refers to the millions of microorganisms that populate an individual’s intestines in a symbiotic relationship. These microorganisms modulate human health by enhancing nutrient metabolism and absorption, maintaining the intestinal epithelial barrier, and promoting host defense and immune homeostasis (Sekirov et al., 2010). Gut dysbiosis refers to an imbalance of the microorganisms in the intestines, and it increases susceptibility to many diseases (Weiss and Hennet, 2017). Infection, other diseases, and antibiotics can cause an imbalance in the microorganisms (Cani et al., 2008; Ivanov and Honda, 2012). The gut microbiota communicates with the central nervous system (CNS) through neural, immune, endocrine, and metabolic pathways, which comprise the gut-brain axis. This axis can regulate gastrointestinal motility and affect emotional and cognitive function. The gut microbiota can modulate brain function and behavior through this axis (Diaz Heijtz et al., 2011; Collins et al., 2012). In AD patients, gut dysbiosis can trigger host systemic immune responses and aggravate inflammatory responses in the brain, contributing to cognitive decline (Zhu F. et al., 2020). Although several reviews have been published on the role of gut microbiota in brain function (such as cognition), there are few reviews on the relationship between gut microbiota and PND. This review aimed to summarize the mechanistic linkage between gut microbiota and PND progression and examine the influence of perioperative factors on gut microbiota, and explore potential interventions related to gut microbiota.

## Perioperative Neurocognitive Disorders

### Origin and Nomenclature

Cognitive decline after anesthesia/surgery is a common clinical phenomenon (Terrando et al., 2011). As early as 1887, Savage first recorded a case of insanity after anesthesia/surgery (Savage, 1887). Bedford (1955) conducted a retrospective study of 251 patients aged >65 years and found that 7% (18/251) developed extreme dementia after general anesthesia. To our knowledge, this is the first official report of postoperative cognitive dysfunction (POCD). Over the past 50 years, numerous studies on POCD have been published. In the 1990s, the International Study of Postoperative Cognitive Dysfunction (ISPOCD) group was established, which conducted basic research and a series of multicenter clinical epidemiological studies on POCD. The ISPOCD reported that the incidence of POCD in elderly individuals was 25.8% at 1 week and 9.9% at 3 months after major non-cardiac surgery (Moller et al., 1998). A study in 2008 demonstrated that the 1-year mortality for patients with POCD within 3 months after surgery was almost twice that of patients without POCD (Fodale et al., 2010).

**FIGURE 1:**
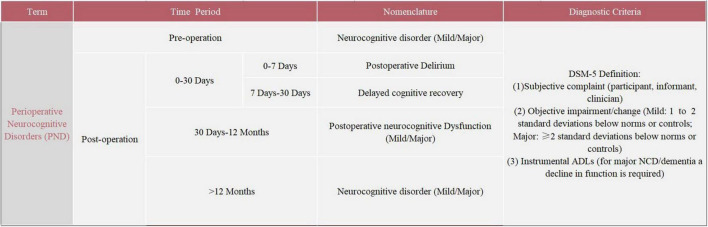
Nomenclature of perioperative neurocognitive disorders (PND).

The severe symptoms and high incidence of POCD deserve serious attention. The evaluation of POCD has mainly relied on a neuropsychological test battery. The neuropsychological tests for POCD used in clinical settings mainly include the Mini-Mental State Examination (MMSE), Montreal Cognitive Assessment (MOCA), Wechsler Adult Memory Scale (WMS), and Wechsler Intelligence Scale (WIS). The absence of specific criteria for the assessment and diagnosis of POCD has led to significant heterogeneity in study results, which seriously limits basic and clinical research on POCD (Evered and Silbert, 2018). In addition, inconsistent with the existing clinical diagnostic criteria for neurocognitive disorders (NCD) in the fifth edition of the Diagnostic and Statistical Manual of Mental Disorders (DSM-5), neuropsychological tests do not take into account the patient’s cognitive functioning and preoperative activities of daily living (ADLs) (Gauthier et al., 2006; Sachdev et al., 2015). This creates serious obstacles to communication between members of different disciplines. In 2018, recognizing the disadvantages of the terminology, the International Perioperative Cognition Nomenclature Working Group (composed of multidisciplinary experts) standardized the nomenclature regarding the cognitive function changes related to anesthesia/surgery. The term “perioperative neurocognitive disorders” (PND) was proposed, which is a comprehensive term to describe impaired cognitive function in the perioperative period, replacing the previous term “postoperative cognitive dysfunction” (POCD) (Evered et al., 2018). PND is divided into the following four categories by time period included: preoperative neurocognitive disorder, POD, delayed neurocognitive recovery, and postoperative neurocognitive disorder (pNCD or POCD). Details regarding PND are summarized in Figure 1. Due to the variability in the previous studies, the term POCD will be used interchangeably with the updated nomenclature throughout this review.

### Risk Factors of Postoperative Cognitive Dysfunction

Several risk factors for POCD were identified, mainly associated with the patient (such as age, preoperative cognitive function level, level of education, and genetic factors), the surgery (such as surgery type), intraoperative complications, and anesthesia (such as anesthesia approaches and anesthetic types). The elderly population is at high risk of POCD following anesthesia/surgery; the proportion of elderly (>60 years of age) with cognitive decline at 3 months after surgery was twice that of middle-aged and young people (12.7% vs. 5.6% vs. 5.7%) (Monk et al., 2008; Silbert et al., 2014). Both clinical and animal studies have indicated that advanced age is an independent risk factor for POCD.

Additionally, cardiac surgery is usually accompanied by a high incidence of POCD, with 50–70% of patients exhibiting cognitive decline 1 week after cardiac surgery and 13% at 1 year (Newman et al., 2006). It was thought that the high incidence of POCD after cardiac surgery may be attributable to the generation of cerebral microemboli during cardiopulmonary bypass (CPB). However, Liu et al. (2009) discovered that the incidence of POCD did not significantly decrease at either 1 week or 3 months after coronary artery bypass grafting without CPB (Kozora et al., 2010; Lamy et al., 2013). This indicates that CPB is not the only causative factor for POCD.

Many studies have attempted to determine the effect of anesthesia or surgery on POCD. A randomized controlled trial of 57 total knee arthroplasty patients showed that patients who received regional anesthesia achieved better neurocognitive test scores than those who received general anesthesia (Edipoglu and Celik, 2019). Nevertheless, Zhang et al. (2015) found no effect of general anesthesia on neuroinflammation and learning memory deficits in aged rats after surgery. Meanwhile, a meta-analysis reported no significant difference in the incidence of POCD between general anesthesia and other anesthesia groups (Mason et al., 2010). Therefore, the effect of the anesthesia method on POCD requires further exploration. The occurrence of POCD is also related to the types of anesthetics. The use of anticholinergic drugs (e.g., atropine and phencyclidine hydrochloride) before general anesthesia increases the incidence of POCD (Plaschke et al., 2013; Rossi et al., 2014). In a mouse model of POCD, pretreatment with an acetylcholinesterase inhibitor improved anesthesia/surgery-induced impairment of working memory (Zhang et al., 2018). Furthermore, the incidence of POCD was higher in elderly patients treated with sevoflurane inhalation anesthesia than those treated with propofol maintenance anesthesia (Qiao et al., 2015; Sun H. et al., 2019). However, the latest research conducted by Li et al. (2021) discovered that the choice of anesthesia between propofol and sevoflurane did not appear to affect the incidence of delayed neurocognitive recovery at 5–7 days after laparoscopic abdominal surgery. Besides, emerging evidence indicates that perioperative dexmedetomidine may decrease the risk of POCD (Zhou et al., 2016; Deiner et al., 2017). Su et al. (2016) demonstrated that low-dose intravenous dexmedetomidine significantly decreased the incidence of POD in elderly patients admitted to intensive care units (ICU) after elective non-cardiac surgery. Conversely, Xu et al., hypothesized that it is surgical trauma, but not anesthesia contributes to the development of POCD and neuroinflammation. They performed abdominal surgery on aged wild-type mice under local anesthesia and found that surgery without general anesthesia could cause cognitive impairment (Xu et al., 2014). The same conclusion also appeared in the study of Lai et al. (2021a). The incidence of POCD varies with different types of surgery, with high incidences after orthopedic surgery such as joint replacement (25–50%), and cardiac surgery (20–50%) (Galanakis et al., 2001). In addition, intraoperative cerebral hypoxia, hypocapnia, cerebral perfusion insufficiency, cerebral embolism, and perioperative hyperglycemia have been reported to be related to the occurrence of POCD (Djaiani et al., 2012; Papadopoulos et al., 2012).

### Pathogenesis of Postoperative Cognitive Dysfunction

The pathophysiological mechanism of POCD remains to be elucidated. The hypotheses regarding POCD mainly include the cholinergic dysfunction hypothesis, β-amyloid (Aβ) cascade hypothesis, tau hyperphosphorylation hypothesis, oxidative stress hypothesis, and inflammation hypothesis. Acetylcholine in the cholinergic CNS is an essential neurotransmitter in the brain. It plays a significant role in the formation and maintenance of learning and memory (Blokland, 1995; Kihara and Shimohama, 2004). This explains why anticholinergics increase the risk of POCD. Cholinergic CNS neurons degenerate with age, decreasing acetylcholine synthesis and release (Zhang et al., 2018). Therefore, it makes elderly individuals prone to POCD. Moreover, inhaled isoflurane may affect cognitive function by inhibiting the transport of acetylcholine in the CNS (Jansson et al., 2004).

It is widely acknowledged that excessive Aβ deposition and tau protein phosphorylation are involved in the pathogenesis of AD. Aβ and hyperphosphorylated tau deposits in the brain can induce mitochondrial damage, synaptic dysfunction, and neuron apoptosis, leading to progressive cognitive impairment (Decker et al., 2010). In animal research, isoflurane exposure increased the levels of Aβ and phosphorylated tau protein in the brain and caused spatial memory impairment (Zuo et al., 2018). Additionally, patients with low preoperative plasma levels of Aβ42 and Aβ40 exhibited cognitive impairment at 3 months after surgery (Evered et al., 2009). These findings suggest that POCD and AD may share a common pathogenic mechanism.

The neuroinflammation theory of POCD has been the focus of recent research. Excessive release of peripheral inflammatory cytokines caused by anesthesia/surgery activates microglia, disturbs the blood–brain barrier (BBB), and allows inflammatory cytokines [interleukin 6 (IL-6), interleukin 10 (IL-10), and tumor necrosis factor α (TNF-α)] to be released into the CNS, thereby inducing neuroinflammation and cognitive impairment (Safavynia and Goldstein, 2018). Biochemical tests of cerebrospinal fluid (CSF) can objectively demonstrate the presence of intracerebral inflammation. Inflammatory cytokines (IL-6, IL-10, and TNF-α) in CSF after anesthesia/surgery are all higher than normal, indicating that postoperative neuroinflammatory reactions do indeed occur in the brain (Tang et al., 2011). There is evidence to suggest that volatile anesthetic isoflurane induces cognitive dysfunction mediated by neuroinflammation in rodents (Cao et al., 2012; Zhang et al., 2014). Similarly, studies have found that neuroinflammation occurs after anesthesia/surgery in humans (Tang et al., 2011). In addition, blocking Kv1.3 potassium channels can prevent postoperative neuroinflammation and cognitive decline in a mouse model (Lai et al., 2020).

Oxidative stress is caused by excessive free radical production and an impaired antioxidant defense system, which is related to mitochondrial dysfunction, hyperphosphorylation of tau protein, Aβ deposition, and neuroinflammation. The superoxide radicals produced during oxidative stress harm neurons and hence contribute to cognitive dysfunction (Ienco et al., 2011). In a rodent model of POCD, malondialdehyde (which reflects the degree and severity of cellular damage by reactive oxygen species) was increased and superoxide dismutase (which reflects the body’s ability to remove reactive oxygen species) was decreased in the hippocampus, indicating that oxidative stress in the brain may be involved in the pathogenesis of POCD (Liu et al., 2019).

Lastly, many recent studies have found that the gut-brain axis may be involved in the mechanism of POCD. The gut microbiota plays a vital role in the development of the CNS. Gut dysbiosis can increase the risk of POCD by damaging the intestinal mucosal barrier and BBB, which can trigger neuroinflammation and oxidative stress in the brain and eventually alter cognitive function (Yang et al., 2018; Zhan et al., 2019).

## Gut Microbiota

Human beings live in a microbial world, with the microbiota affecting the development of most organ systems and regulating metabolism. Microbes can colonize multiple sites in the body, particularly the skin, eyes, mouth, respiratory tract, urogenital tract, and gastrointestinal tract (Bäckhed et al., 2005; Artis, 2008; Belkaid and Hand, 2014). Nevertheless, most microbes in humans live in the gastrointestinal tract. The human gastrointestinal tract harbors about 10^13^–10^14^ microorganisms, approximately 10 times the number of human cells in the body, and the combined genetic material of the gut microbiota (i.e., the gut microbiome) comprises more than 100 times the number of genes in the human genome (Eckburg et al., 2005; Lozupone et al., 2012). The gut microbiota which is composed of bacteria, viruses, archaea, protists, and fungi (including yeasts), is a highly complex and densely populated ecosystem. Trillions of gut microbes participate in physiological and pathophysiological processes in the body that affect host health throughout the lifespan (Lim et al., 2016; Heiman and Greenway, 2016). Therefore, the gut microbiota has been referred to as a “forgotten organ” (O’Hara and Shanahan, 2006). The gut bacteria are characterized by a wide diversity of species, divided into six main phyla: *Firmicutes, Bacteroidetes, Proteobacteria, Actinomycetes, Verrucomicrobia*, and *Fusobacteria*. Among them, Firmicutes (such as *Clostridium, Enterococcus, Lactobacillus, and Ruminococcus*) account for 60% of the gut microbiota, and Bacteroidetes (such as *Bacteroides and Prevotella*) accounts for 15% (Eckburg et al., 2005; Lozupone et al., 2012). The complexity and diversity of the gut microbiota are established in infancy and are influenced by external factors such as childbirth (vaginal or Cesarean), breastfeeding or formula feeding, weaning, antibiotics, infection, diet, and stress (Drell et al., 2017). The gut microbiota of the newborn depends on the mode of delivery, with vaginal delivery leading to a highly similar gut microbiota to the maternal vaginal microbiota. In addition, breastfed infants have a more complex *Bifidobacterium* microbiota community than formula-fed infants (Rinne et al., 2005). With the addition of complementary food, the gut microbiota stabilizes to produce a more adult-like profile around the age of 2 years. The adult-like gut microbiota is considered to be relatively stable throughout adulthood, although it is vulnerable to the influences of antibiotics, diet, stress, and lifestyle, and it changes somewhat with age (Charbonneau et al., 2016). The gut microbiota of the elderly compared to the young is characterized by decreased bacterial diversity, changes in the dominant species, decreased beneficial microorganisms, increased facultative anaerobic bacteria, and decreased production of short-chain fatty acids (SCFAs). These changes may be related to the diet and other lifestyle factors of the elderly (Salazar et al., 2014).

## Gut-Brain Axis

The gastrointestinal tract is the largest digestive and immune organ in the human body. It contains about 500 million nerve endings, which form the enteric nervous system (ENS). About 20% of them are classified as endogenous primary afferent neurons, which transmit subtle changes in the gastrointestinal tract to the brain via the vagus nerve (Cani and Knauf, 2016). When the host suffers from inflammation or infection, immune cells release important cytokines, and neuroendocrine hormones (such as cortisol) change the intestinal permeability, penetrate the intestinal mucosal barrier and BBB, and communicate with cytokines secreted by immune cells, thus affecting the function of the intestinal tract and brain (Carabotti et al., 2015; Obrenovich, 2018). Based on the bidirectional communication between the gut microbiota and the CNS, the concept of the gut-brain axis was proposed. There are multiple communication routes related to this axis, including the neuroanatomical pathway, the hypothalamic-pituitary-adrenal (HPA) axis neuroendocrine pathway, the immune system, the gut microbiota metabolism pathway, and the intestinal mucosal barrier and BBB (Wang and Wang, 2016). Achieving two-way communication between the brain and the gut requires the cooperation of multiple systems, including the ENS [a subdivision of the autonomic nervous system (ANS)], CNS, immune system, and endocrine system. Numerous neurotransmitters, such as dopamine, γ-aminobutyric acid (GABA), and serotonin [5-hydroxytryptamine (5-HT)], are involved in the bidirectional communication between the gut and the brain. Germ-free (GF) mice (without gut microbiota) have abnormal brain function, which can manifest as learning disabilities, anxiety-like behavior, and decreased social skills. These behaviors may be related to changes in the amygdala and hippocampus, as their volume and dendritic morphology were significantly different between GF and normal mice, including shorter neurites, a lower branching degree, and a thinner spinal cord (Luczynski et al., 2016; Trzeciak and Herbet, 2021). In addition, the gut microbiota affects myelination and neurogenesis in the adult brain by regulating microglial activation, thus affecting the inflammatory response in the brain (Erny et al., 2015; Hoban et al., 2016). In turn, the brain regulates intestinal movement, intestinal secretion, and immune function, with neural circuits, neurotransmitters, and receptors being involved in the physiological regulation of intestinal function (Jacobson et al., 2021). When neurotransmission is abnormal, the HPA response can change and intestinal neurons can be damaged, potentially leading to an abnormal microbial community. For example, PD patients often have a high incidence of gastrointestinal dysfunction (Cersosimo and Benarroch, 2012).

## Gut Microbiota and Cognition

Cognition is the process by which the brain receives external information, processes it, and converts it into internal mental activities to acquire or apply knowledge. It includes aspects such as memory, language, visuospatial skill, execution, calculation, understanding, and judgment (Le Pelley et al., 2016). Cognitive impairment refers to abnormal changes or functional decline in these cognitive functions in the higher center of the brain. Many factors have been shown to affect cognitive function, including stress, diet, genetics, infection, and inflammation (Speisman et al., 2013; Cai et al., 2014; Fan et al., 2017). In recent years, gut microbiota has emerged as a significant factor in the development and maintenance of cognitive function. Many studies have explored the influence of the gut microbiota on host behavior and cognitive function. Cattaneo et al. (2017) showed that gut dysbiosis in patients with cognitive impairment is characterized by a decreased abundance of anti-inflammatory bacteria (*Eubacterium rectale*) and an increased abundance of pro-inflammatory bacteria (*Escherichia* and *Shigella*). Additionally, chronic *Helicobacter pylori* infection can trigger the release of inflammatory factors and Aβ accumulation in AD patients and aggravate the symptoms of cognitive dysfunction (Roubaud-Baudron et al., 2012). Certain gut bacteria (e.g., *Bacteroides vulvae, Bifidobacterium bifidum, Lactobacillus salivarius*, and *Clostridium clusters*) may affect cognitive function in rodents and humans by causing neuroinflammation. Fecal microbiota transplantation (FMT) from transgenic mice with dementia to cognitive healthy mice significantly decreased the cognitive performance of the recipients. This indicates that the gut microbiota plays a critical role in physiological and pathological processes related to cognitive impairment (Zhan et al., 2019).

### Intestinal Mucosal Barrier and Blood–Brain Barrier

The BBB is a highly selective semipermeable membrane that prevents circulating toxins and pathogens from accessing the brain while allowing important nutrients and oxygen to enter. The BBB is composed of endothelial cells that are closely connected together, forming “tight junctions.” It plays a pivotal role in homeostasis in the brain. When it is damaged, peripheral toxic substances enter the brain via the bloodstream, which poses serious threats to the brain (Obermeier et al., 2013). BBB dysfunction has been shown to be associated with many neurodegenerative diseases, such as AD, multiple sclerosis, and neuromyelitis optica (Acharya et al., 2013). In AD patients, cognitive impairment, which is mainly manifested in the hippocampus, is related to BBB damage. The BBB leakage rate was higher in patients with early AD than healthy controls, based on dynamic contrast-enhanced magnetic resonance imaging. This suggests that the breakdown of the BBB may be a key mechanism in the early stage of AD (Zenaro et al., 2017; Sweeney et al., 2018). In addition, traumatic brain injury can also lead to severe BBB damage, increasing the risks of cognitive impairment and dementia (Lye and Shores, 2000).

Research on specific-pathogen-free (SPF) mice has shown that the BBB develops around the second week of embryo development, with a sharp decrease in BBB permeability after embryonic day 15. In contrast, the BBB permeability of GF mice continued to increase after embryonic day 15 and into adulthood. Thus, it can be seen that microorganisms influence the development of the BBB in the embryonic stage (Sampson and Mazmanian, 2015). In GF mice compared to pathogen-free mice, BBB permeability was increased in various brain regions (including the frontal cortex, hippocampus, and striatum). Furthermore, transferring feces from pathogen-free mice to GF mice or treating GF mice with bacteria that produce SCFA reduced BBB permeability (Braniste et al., 2014). Recent studies have shown that the gut microbiota can improve postoperative cognitive function in aged mice by reducing the BBB permeability (Wen et al., 2020).

The intestinal barrier is composed of a polarized monolayer of epithelial columnar cells that are tightly bound together by intercellular tight junctions. These junctions are composed of four types of integral membrane proteins: occludin, claudins, tricellulin, and junctional adhesion molecule, which regulate paracellular permeability and ensure the intestinal epithelial barrier integrity (Furuse et al., 1993; Tsukita et al., 2001). The function of the intestinal barrier is to regulate the balance of nutrients, water, and electrolytes and to prevent pathogens and toxic substances from entering the systemic circulation from the intestinal lumen. If the intestinal barrier is damaged and the intestinal wall becomes permeable, pathogens (bacteria, viruses, and fungi), toxins, and incompletely digested food molecules can pass through the barrier into the bloodstream and reach the brain, causing astrocyte swelling and pro-inflammatory factor release in the brain. The gut microbiota plays a key role in intestinal barrier homeostasis (Farhadi et al., 2003). Antibiotics cause *Clostridium difficile* reproduction and colonization of the intestinal epithelium. *C. difficile* produces toxins that impair actin filament aggregation, damage tight junctions and increase intestinal permeability (Hecht et al., 1988). In contrast, healthy and mature gut microbiota can maintain tight junction protein structure and inhibit intestinal inflammation. Animal studies showed that changes in the gut microbiota composition can lead to the breakdown of the intestinal epithelial barrier and increased mucosal permeability, with the translocation of gut bacteria to the whole body, which contributes to Toll-like receptor 4 (TLR4) activation and the initiation of inflammatory processes in the brain (Alhasson et al., 2017). This process may be involved in the development of progressive cognitive impairment and dementia.

### Intestinal Immune System

Mounting evidence suggests that there are complex interactions between the gut microbiota and the host immune system. The gut microbiota can regulate the development and function of innate and adaptive immune systems and maintain the dynamic balance in the immune system. In turn, the innate and adaptive immune systems can promote gut microbiota and host homeostasis (Yoo et al., 2020). Additionally, the indirect effects of gut microbiota on the innate immune system may change the levels of circulating cytokines that directly affect brain function (Rogers et al., 2016). The release of pro-and anti-inflammatory cytokines is involved in the development of brain diseases such as AD, depression, and autism. Microglia, which are the innate immune cells of the CNS, regulate CNS development and maintenance and play an immune surveillance role in the brain, participating in information transmission and clearing cell fragments (Legroux and Arbour, 2015). The gut microbiota plays a crucial role in shaping the maturation and homeostasis of microglia. The innate immune response of microglia in GF mice is highly weakened, suggesting that the existence of the gut microbiota promotes microglial maturation and increases resistance to challenges by bacteria and viruses. The microglial function can be partially restored by gut microbiota transplantation (Erny et al., 2015).

Lipopolysaccharide (LPS), also known as endotoxin, is a major component of the outer membrane of Gram-negative bacteria. The gut microbiota is an important source of LPS and Aβ. LPS can stimulate the host immune system to damage intestinal epithelial cells and it can thereby access the blood circulation. LPS can then activate microglia and promote neuroinflammation in the brain, triggering cognitive decline (Banks et al., 2015; Sorrenti et al., 2018). LPS has been observed in the hippocampus and neocortex of patients with AD. Intraperitoneal LPS injection in mice increased the hippocampal level of Aβ42 and impaired cognitive function (Kahn et al., 2012; Zhao et al., 2017). LPS promotes the expression of its receptors TLR4 and CD14, which enhances inflammatory cytokine release and Aβ production in the brain. Aβ can also activate TLR4, continuously increasing Aβ levels in the brain and aggravating the progression of AD (Kapil et al., 2016; Yang et al., 2020).

### Hypothalamus-Pituitary-Adrenal Axis

The HPA axis is an important part of the gut-brain axis, and it is involved in controlling stress responses and regulating many physical activities, such as digestion, the immune system, mood and emotion, sexual behavior, and energy storage and consumption (Fumagalli et al., 2007). When a stress response occurs, the paraventricular nucleus in the hypothalamus synthesizes and releases corticotropin-releasing hormone (CRH), which stimulates the release of adrenocorticotropic hormone (ACTH) in the pituitary gland. ACTH stimulates the adrenal cortex to release the end product cortisol (Dunn, 2000; Charmandari et al., 2005). Cortisol receptors are distributed in multiple regions of the CNS, including the hippocampus, hypothalamus, and amygdala. When cortisol concentrations are elevated, cortisol-mediated activation of cortisol receptors in the hippocampus and hypothalamus exerts negative feedback on HPA activity, thereby terminating the stress response (De Kloet et al., 1998; Pruessner et al., 2003). High cortisol levels associated with acute stress can cause neuronal amyloidosis and tau phosphorylation, thereby affecting cognitive function (de Kloet et al., 1999; Green et al., 2006). In addition, clinical research has demonstrated that AD patients with cognitive impairment have concomitant HPA axis dysfunction, mainly characterized by elevated cortisol levels and abnormal cortisol receptor expression (Aznar and Knudsen, 2011). There is a close mutual relationship between the HPA axis and gut microbiota. The stress response may cause gut dysbiosis and increased intestinal permeability (Kelly et al., 2015). GF mice exhibit an excessive increase in HPA axis activity in response to stress, manifested as increased levels of cortisol and corticotropin. Probiotic supplementation can ameliorate stress-induced HPA axis dysfunction and improve cognitive impairment as well as depressive- and anxiety-like symptoms (Sudo et al., 2004; Liang et al., 2015).

Additionally, LPS activates the HPA axis, increasing the stress hormone levels, including cortisol, which can affect cognition (Grinevich et al., 2001). Patients with cognitive impairment have higher levels of cortisol in the CSF than normal people (Comijs et al., 2010; Ouanes and Popp, 2019). Moreover, the increase of serum cortisol level can change the gut microbiota composition and intestinal barrier permeability (Farzi et al., 2018). And gut dysbiosis caused by antibiotics increased the serum corticosteroid level and caused neuroinflammation and cognitive-behavioral abnormalities in rats (Wang et al., 2015).

### Gut Metabolism System

The major function of the gut microbiota for humans is to digest food. The gut microbiota converts carbohydrates into SCFAs. SCFAs can activate G protein-coupled receptors to exert neuroprotective effects. SCFAs can also restore the memory function of mice with AD by inhibiting histone deacetylase. Moreover, SCFAs can prevent AD by interfering with the assembly of Aβ peptides into neurotoxic polymer. Furthermore, SCFAs can regulate microglia homeostasis, suppress demyelination, and enhance remyelination in the prefrontal cortex, which is responsible for complex cognitive tasks such as planning and decision-making (Ho et al., 2018; Silva et al., 2020; Wenzel et al., 2020).

In addition to absorbing and producing metabolites, the gut microbiota can synthesize intestinal toxins (such as LPS), and also neurotransmitters (such as GABA, 5-HT, and DA), and vitamins (such as vitamins K and B), which are essential for brain function and behavior (Fung et al., 2017; Sherwin et al., 2018). *Lactobacillus* and *Bifidobacterium* can participate in the synthesis of endogenous GABA, which is an inhibitory neurotransmitter in the CNS. About 20–30% of the CNS synapses employ GABA as a neurotransmitter (Watanabe et al., 2002). Anesthesia and surgery disrupted the GABAergic system and contributed to hippocampus-dependent memory and cognitive dysfunction, which may be related to the P38 MAPK signaling pathway (Zhang et al., 2020). Additionally, recent studies showed that the inverse agonist of the α5 subunit-containing GABAA (α5GABAA) receptor, 5IA, attenuated the Aβ-induced death of hippocampal neurons and enhanced cognitive function (Vinnakota et al., 2020).

5-hydroxytryptamine, which is converted from tryptophan, plays important roles in neuronal and glial development, which are related to cell proliferation, differentiation, migration, apoptosis, and synapse formation (Gaspar et al., 2003). Gut microorganisms in mice synthesize up to 60% of the 5-HT present in the colon and blood. The gut microbiota can also induce pheochromocytes in the intestinal epithelium to synthesize 5-HT, generate and release metabolites, stimulate immune cells to secrete cytokines, regulate nerve cells to affect brain activity, and ultimately affect human mood, learning, memory, and behavior (Roth et al., 2021).

Vitamin K is a fat-soluble vitamin that is involved in the synthesis of sphingolipids, which are an important component of the CNS cell membrane and are associated with the proliferation and differentiation of neurons. Changes in sphingomyelin expression are related to neurodegeneration and neuroinflammation (Denisova and Booth, 2005; Ferland, 2012). Vitamin K can also inhibit Aβ deposition by activating growth arrest-specific gene 6 (Gas-6), which has a protective effect on neurons. Low vitamin K intake or serum concentration is directly related to cognitive impairment in the elderly aged ≥65 years (Yagami et al., 2002; Tamadon-Nejad et al., 2018).

The B vitamins are vital dietary components. An increased level of homocysteine, which can be lowered by increased vitamin B intake, is linked to cognitive dysfunction. The level of vitamin B is decreased in AD patients. Vitamin B supplementation improves memory and slows down the process of cognitive decline (Seshadri et al., 2002; Clarke et al., 2014).

### Vagus Nerve Pathway

The regulation of the gastrointestinal nervous system involves the CNS and ENS (a subdivision of the ANS). The ENS is a complex autonomic neural network composed of sensory, motor, and intermediate neurons that can independently regulate the basic functions of the gastrointestinal tract (movement, mucus secretion, and blood circulation). The ENS can transmit the information sensed by the intestinal tract directly to the brain via its intestinal nerves (branches of the ANS) and vagal afferent nerves (Obata and Pachnis, 2016). In fact, the vagus nerve is the main neural communication route between the gut microbiota and the brain. The ends of the vagal afferent nerves are located in the intestinal mucosa where a large number of intestinal regulatory peptides and receptors for intestinal metabolites are distributed (Bonaz et al., 2018). Bacterial neurotransmitters and neuropeptides can directly activate the intermuscular neurons and transmit signals to the brain via the vagal afferent nerves. Changes in the gut microbiota composition can directly alter mood and cognition via the vagus nerve (Pellegrini et al., 2018). Transplantation of *Campylobacter jejuni* into the gut of mice induced anxiety-like behavior, while transplantation of non-pathogenic bacteria (such as *Lactobacillus rhamnosus*) into the duodenum had anti-anxiety and antidepressant effects, but required an intact vagus nerve (vagotomy prevented the effects of *L. rhamnosus*) (Bravo et al., 2011). Yun et al. (2020) discovered that *Lactobacillus gasseri* NK109 alleviated *Escherichia coli*-induced cognitive impairment by modulating IL-1β expression, gut microbiota, and vagus nerve-mediated gut-brain signaling. Nonetheless, the mechanism underlying the interaction between intestinal microorganisms and the vagus nerve remains to be elucidated.

## Gut Microbiota in the Perioperative Period

### Preoperative Period

The perioperative period usually includes the periods related to hospitalization, anesthesia, surgery, and rehabilitation. The number of elderly people needing surgery has increased, which brings great challenges to every component of the perioperative period. The diversity of gut microbiota changes with age. Elderly people tend to have a reduced diversity of gut microbiota compared to young people, characterized by lower levels of *Firmicutes* and *Actinobacteria* and higher levels of Proteobacteria (Hopkins and Macfarlane, 2002; Rondanelli et al., 2015). Additionally, the serum level of SCFA, the main bacterial metabolite in the colon, is lower in the elderly than in the young. These changes may be related to the low dietary absorption rate and decreased immune function in the elderly (Biagi et al., 2010). Preoperative fasting and fluid limitation are routine in the perioperative period. However, diet is a pivotal determinant in the gut microbiota community structure and function, so fasting may trigger gut microbiota changes that may in turn affect host health and immunity (Jabbar et al., 2003; Carmody et al., 2015). In animal models, the gut microbiota changed rapidly within 1–3 days of fasting. With increased fasting duration, the structure of the jejunal microbiota changed significantly, with decreases in β*-bacilli* and *Bacteroides*. Surgical patients are often exposed to preoperative psychological stress (such as anxiety and fear) and/or physiological stress (such as insomnia and malnutrition) (Wetsch et al., 2009; Kohl et al., 2014). These stress stimuli can activate the sympathetic nervous system and HPA axis, thus increasing intestinal permeability and inflammation, and ultimately affecting the gut microbiota. Psychological stress has been reported to alter the microorganism colonization of the mucosal surface and the host’s susceptibility to infection (Bailey et al., 2011; Vanuytsel et al., 2014).

### Intraoperative Period

A recent study showed that general anesthesia negatively alters the diversity and composition of the gut microbiome. Ma et al. found that 4-h exposure to a volatile anesthetic (isoflurane) in mice significantly decreased the microbial diversity and the levels of several commensal bacteria including *Clostridiales*. Thus, volatile anesthetics are potential contributors to gut dysbiosis in postoperative patients (Serbanescu et al., 2019). Opioid analgesics are the most commonly used medication for the management of postoperative pain, but they increase susceptibility to intestinal infection by *C. difficile*, *Vibrio cholerae*, *Salmonella enterica*, and *Pseudomonas aeruginosa* (Mora et al., 2012). Another study revealed that morphine can damage the intestinal epithelial barrier function and increase the translocation of gut microbiota in mice (Meng et al., 2013).

Surgical interventions, especially gastrointestinal surgery, threaten the balance of gut microbiota in patients. Gastrointestinal surgery often involves intestinal short circuit and anastomosis construction, which directly changes the habitat of the gut microbiota (Aron-Wisnewsky and Clement, 2014; Wang and Qin, 2020). The diversity and abundance of gut microbes in patients with gastric cancer undergoing surgical treatment were found to be increased. Additionally, gastrectomy increased the abundance of aerobic bacteria, facultative anaerobic bacteria, and oral microbes, which may be related to the digestive tract reconstruction and/or postoperative complications (Erawijantari et al., 2020). A meta-analysis of the relationship between the gut microbiota and postoperative complications suggested that surgery often increased potential pathogens such as *Pseudomonas, Staphylococcus*, and *Enterococcus, and* decreased *Lactobacillus* and *Bifidobacterium* (Lederer et al., 2017). To prevent postoperative infection, perioperative antibiotic use is key. However, many studies have shown that antibiotics can lead to short- or long-term effects on the gut microbiota in humans and animals, such as changes in the composition of the gut microbiota, changes in diversity, and delays in colonization time (Francino, 2015). In particular, broad-spectrum antibiotics can seriously damage the gut microbiota and lead to diarrhea, which may be related to excessive *C. difficile* growth or reduced SCFA production. In addition, by interfering with the gut microbiota, antibiotics can affect drug metabolism and decrease the body’s resistance to external pathogens, thus increasing the risk of infection (Kim, 2015; Abt et al., 2016).

### Postoperative Period

Postoperative complications are closely related to the gut microbiota, with the gut microbiota playing an important role in preventing pathogens from crossing the intestinal barrier. Additionally, the composition of the gut microbiota could reflect the response efficiency of the immune system to invasive pathogens (Schmitt et al., 2019). A systematic review conducted by Lederer et al. (2017) demonstrated that changes in the gut microbiota after gastrointestinal surgery may be associated with the development of postoperative complications such as wound infections or anastomotic leakage. In a pilot study involving 26 patients who underwent renal transplantation, the gut microbiota in the fecal samples collected at 3 months after renal transplantation surgery exhibited significant changes, which was related to complications such as diarrhea, acute rejection, and urinary tract infection (Lee et al., 2014). The most common postoperative complication is pain, such as visceral pain after gastrointestinal surgery. Gastrointestinal surgery induces gut microbiota disruption, intestinal barrier damage, and intestinal inflammation, which may contribute to visceral pain, with sensitization of the peripheral and central pain pathways. Visceral pain linked to changes in gut microbiota diversity and abundance was improved after normalizing the gut microbiota (Chichlowski and Rudolph, 2015; Pusceddu and Gareau, 2018). In summary, the gut microbiota influences the patient throughout the perioperative period.

## Gut Microbiota and Perioperative Neurocognitive Disorders

### Possible Roles of Gut Microbiota in Perioperative Neurocognitive Disorders Development

There are several hypotheses about the mechanisms underlying PND, including a hypothesis involving the gut microbiota. Few studies are focusing on the relationship between the gut microbiota and PND (Table 1), though this relationship is becoming a research hotspot. Many perioperative factors can cause gut dysbiosis. The abundance of gut microbiota has been shown to change significantly after surgery/anesthesia, with an increase in the proportion of Gram-negative bacteria. LPS, a key component of Gram-negative bacteria cell walls, is a strong agonist of TLR4 on the surface of intestinal epithelial cells, increasing intestinal permeability (Guo et al., 2013). Additionally, surgical trauma and oxidative stress caused by surgery/anesthesia can induce the release of proinflammatory factors such as IL-1β, IL-6, and TNF-α (Wakabayashi et al., 2014). Proinflammatory cytokines can damage the integrity of the BBB and cross the BBB via specific surface receptors and transporters on BBB endothelial cells, eventually contributing to microglial activation and neuroinflammation in the brain. The gut microbiota can synthesize and release neurotransmitters such as 5-HT, GABA, and dopamine. Accumulating evidence indicates that delirium results from neurotransmitter system dysfunction (Li et al., 2012). There were significantly increased concentrations of dopamine and 5-HT metabolites in the CSF, hippocampus, and basolateral amygdala of rats with POD. After treatment with the selective 5-HT1A antagonist WAY-100635, the rats showed a partial reversal of the POD symptoms. This may be attributable to the inhibition of PI3K/Akt/mTOR activation in the hippocampus and basolateral amygdala by the treatment, thus hampering NLRP3-mediated release of IL-1β into the CSF (Qiu et al., 2016). GABA subtype A (GABAA) receptors are important targets for most anesthetics. The α5GABAA receptor of non-vertebral cells in the hippocampus was shown to be activated by the anesthetic etomidate, which was associated with the anesthesia-induced amnestic effect and memory decline (Cheng et al., 2006; Zurek et al., 2014). Moreover, in rats, the GABAA receptor is critical for regulating cognition, including influencing spatial learning by activating the protein kinase A signaling pathway (Cheng et al., 2006; Zurek et al., 2014). After anesthesia/surgery, there has been reported to be an increased proportion of *E. coli* in the gut. *E. coli*, *Bacillus subtilis*, and *Salmonella* in the gut can synthesize Aβ protein, which disrupts the host’s defense system by enhancing pathogenic microorganism adhesion and biofilm development (Friedland and Chapman, 2017). Intestinal Aβ can enter the circulation via a damaged intestinal barrier, thereby triggering cross-reactive immune responses. This excessively activates inflammatory signaling and causes Aβ deposition in the brain. Aβ deposition can interfere with the N-methyl-D-aspartate (NMDA) receptor mRNA expression in hippocampal neurons and reduce synaptic plasticity, eventually leading to cognitive impairment (Parihar and Brewer, 2010; Newcombe et al., 2018). In this regard, perioperative intestinal dysbiosis can impair neurotransmitter and neuromodulator synthesis and secretion, causing cognitive dysfunction via the gut-brain axis.

**TABLE 1:** Characteristics of included studies on the relationship between gut microbiota and PND.

Studys	Country	Animal	Surgery	Intervention	Associations found
Wen et al., 2020	China	210 SPF male C57BL/6J mice 90 young (age, 6 weeks; body weight, 25 ± 2 g) and 120 aged (age, 18 months; body weight, 44 ± 2 g)	Splenectomy surgery	*Lactobacillus* mix, oral gavage, 0.2 mL twice a day for 1 month	*Lactobacillus* and NaB protected the postoperative cognitive functions of the aged and gut dysbiosis mice
Liufu et al., 2020	United States	9 or 18 months old female mice	Laparotomy	*Lactobacillus salivarius*, once per day for 10 days before surgery. Probiotics once per day for 20 days before surgery	Microbiota dysbiosis contribute to postoperative delirium and treatment with *Lactobacillus* or a probiotic could mitigate postoperative delirium
Jiang et al., 2019	China	18-month-old C57BL/6 mice	Tibial fracture internal fixation	VSL#3, oral gavage, once a day for 17 days	Deficits in reference memory induced by anesthesia/surgery are mediated by intestinal dysbacteriosis
Feng et al., 2017	United States	Male LCR or HCR rat	Tibial fracture intramedullary fixation	Preoperative exercise 5 days per week for 6 weeks	Exercise can ameliorate the decline of cognition postoperatively by improving diversity of the gut microbiome in the LCR rats
Meng et al., 2019	China	Aged male F344xBN F1	Laparotomy surgery	TMAO (120 mg/kg), 3 weeks (2 weeks preoperatively, 1 week postoperatively)	Elevated circulating TMAO may contribute to exaggerations of neuroinflammation and cognitive decline in aged rats following surgery
Yang et al., 2018	China	SPF Sprague-Dawley male rats (8 months old)	Abdominal surgery with laparotomy combined with mesenteric ischemia-reperfusion	B-GOS solution, 21 days (18 days preoperatively, 3 days postoperatively)	B-GOS has a beneficial effect on regulating neuroinflammatory and cognitive impairment and was associated with gut microbiota
Zhan et al., 2019	China	18-month-old male C57BL/6J mice (28–32 g)	The intramedullary fixation for tibial fracture surgery	Not mentioned	Alterations in the composition of gut microbiota are probably involved in the pathogenesis of POCD in aged mice
Zhang et al., 2019	China	8-week-old C57BL/6J male mice (25 g)	Laparotomy	Transplant fecal bacteria into antibiotics–induced pseudo–germ–free mice	Abnormal gut microbiota composition after abdominal surgery may contribute to the development of POD.
Liang et al., 2018	China	6–8-week-old CD-1 male mice	Exploratory laparotomy	10 mg cefazolin in 0.1 m, intraperitoneally injected 30 min before surgery, once every day for 5 days	Cefazolin can attenuate surgery-induced postoperative memory and learning impairment by affecting gut microbiome in mice
Xu et al., 2021	China	Adult (8–10 weeks old) male C57BL/6J mice	Partial hepatectomy	SCFA mixture orally for 4 weeks	Pretreatment with SCFAs attenuated cognitive impairment induced by surgical trauma and anesthesia
Yu et al., 2019	China	10-week-old SD rats weighing 280–300 g	Ischemia/reperfusion of the left coronary artery	Probiotic once a day, gavage for 2 weeks until the day of surgery	Probiotics may attenuate cognitive impairment caused dysbiosis of the gut flora
Fonken et al., 2018	United States	Adult (3 months) and aged (24 months) male F344XBN F1 rats	Laparotomy surgery	Subcutaneous injections of *Mycobacterium vaccae*, one injection per week for 3 weeks	*Mycobacterium vaccae* mitigate the neuroinflammatory and cognitive impairments induced by surgery

### Prevention and Treatment of Perioperative Neurocognitive Disorders From the Perspective of Gut Microbiota

Perioperative neurocognitive disorders is directly related to prognosis in surgical patients. Therefore, the treatment and prevention of PND are of great significance. Currently, a variety of agents are used to improve the cognitive function of patients with cognitive impairment, including vitamins, synthetic GABA derivatives (*piracetam*), ergot alkaloids (*dihydroergotamine alkaloids*), calcium antagonists (*nimodipine*), cholinesterase inhibitors (*donepezil*), glutamate receptor antagonists (*memantine*), and neurotrophic factors (*nerve growth factors*), but the effects of these agents still need to be confirmed (Whitaker et al., 2002).

Prevention is the best approach for dealing with POCD. Early identification and management of potential perioperative risk factors play an important role in the prevention of POCD. Prolonged preoperative fasting can not only cause gut dysbiosis, but it can also increase the stress response and worsen catabolism, thereby increasing the risk of cognitive impairment (Kotekar et al., 2018; Yang et al., 2019). Hence, the current guidelines recommend shortening the fasting time and carbohydrate pre-loading, which reduces neuroendocrine stress, catabolism, and insulin resistance, and improves patient outcomes (Batchelor et al., 2019). Emerging evidence indicates that the use of antibiotics alone may induce cognitive dysfunction via gut dysbiosis in mice without surgery. However, a study by Liang et al., found that perioperative use of cefazolin could attenuate surgery-induced postoperative memory and learning impairment in mice. This may be related to the direct anti-inflammatory effect of cefazolin (Liang et al., 2018). Besides, Feng et al. (2017) discovered that although the low capacity runner (LCR) rats exhibited cognitive decline following surgery, preoperative exercise improved both the diversity of gut microbiota and cognitive function. Meanwhile, Lai et al. (2021b) found that low-intensity exercise stabilized gut microbiota and reduced the production of valeric acid (a product of gut microbiota harmful to learning and memory), thereby reducing learning and memory impairment in aged mice undergoing surgery. A large amount of clinical evidence indicates that moderate physical activity and social participation can ameliorate cognitive dysfunction (Heyn et al., 2004). Hypothermia can contribute to changes in the composition of the gut microbiota. Perioperative hypothermia increases the risk of infections, delays wound healing, and increases the risk of POCD (Salazar et al., 2011; Madrid et al., 2016). Therefore, temperature monitoring and heating devices should be used to maintain normal body temperature.

Additionally, maintaining the appropriate depth of anesthesia and cerebral oxygenation can reduce the risk of developing POCD (Kotekar et al., 2018). Anesthesia approaches and anesthetic types are also closely related to the occurrence of POCD, and recent studies have shown that they can alter the gut microbiota composition. Regional anesthesia is associated with a lower incidence of POCD than general anesthesia. Moreover, the incidence of POCD after major surgery was higher in patients who received inhalational anesthesia than those maintained with intravenous propofol, while continuous intravenous dexmedetomidine infusion reduced the incidence of POCD (Carr et al., 2018). Therefore, to prevent POCD, regional anesthesia should be considered as an alternative to general anesthesia when appropriate. Furthermore, inhalation anesthesia should be avoided as far as possible and dexmedetomidine is recommended for conscious sedation and as an adjunct for anesthesia. In addition, the use of multimodal analgesia may reduce the incidence of POCD, as the gut microbiota is involved in the regulation of pain, and pain can in turn lead to gut dysbiosis, and pain is also closely linked to POCD (Wang et al., 2007; Guo et al., 2019).

Due to the close relationship between gut microbiota and cognition, several studies conducted to develop agents for cognitive-related diseases have focused on the gut microbiota. The main goal is to restore the abundance and diversity of the gut microbiota. Pretreatment of aged mice with mixed probiotics (VSL#3) or a combination of antibiotics to eliminate the gut microbiota mitigated anesthesia/surgery-induced impairment in reference memory, which was related to alterations in the abundance of 37 bacterial genera (18 families) in the gut (Jiang et al., 2019). Similarly, treatment of mice undergoing anesthesia/surgery with *Lactobacillus* or other probiotics ameliorated anesthesia/surgery-induced changes such as age-dependent POD-like signs, dysbiosis, and synaptic loss, mitochondrial dysfunction, and increased IL-6 in the brain (Liufu et al., 2020). Furthermore, fecal microbiota transplantation (FMT) is a recently proposed treatment for gut dysbiosis. It involves transplanting an ideal donor’s microbiota to supplement or replace the recipient’s gut microbiota. Sun J. et al. (2019) recently reported that FMT improved cognitive impairment in an APPswe/PS1dE9 transgenic mouse model of AD, accompanied by reduced Aβ deposition in the brain and decreased phosphorylation of tau protein. However, this conclusion needs to be further explored and verified in animal models of PND.

## Conclusion and Prospect

**FIGURE 2:**
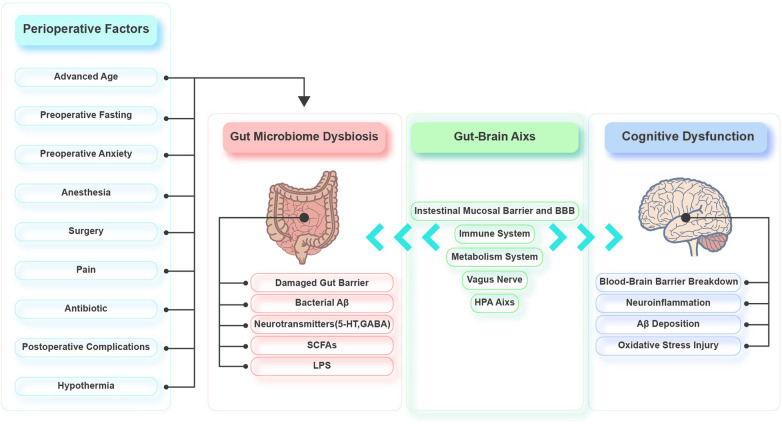
Possible role of gut microbiota in perioperative neurocognitive disorders (PND).

Emerging evidence demonstrates that the gut microbiota is involved in the pathophysiology of PND by regulating the gut-brain axis. Various perioperative factors may affect the diversity and composition of the gut microbiota, causing an imbalance. The interactions between the gut microbiota and the brain are mainly realized through the intestinal mucosal barrier and BBB, immune system, HPA axis, metabolic system, and vagus nerve. Gut dysbiosis damages the intestinal epithelium, resulting in mild chronic inflammation of the intestine and elevated intestinal permeability. Toxic substances can then leak into the circulation, which leads to systemic inflammation and oxidative stress. Systemic inflammation triggers CNS inflammation via the BBB. The CNS inflammation leads to excessive activation of hippocampal microglia, decreased hippocampal synaptic plasticity, and increased hippocampal oxidative stress, neuronal apoptosis, mitochondrial dysfunction, and Aβ deposition (Figure 2). There is little basic research on the gut microbiota and perioperative cognition, and most of the studies lack in-depth exploration. Therefore, mechanistic studies should be conducted to identify specific microorganisms and/or signaling pathways that affect perioperative cognition to identify targets that could be used to develop treatments for PND. Moreover, there are few clinical studies on PND and gut microbiota, and we should be cautious about extrapolating the results of animal studies to the clinic. So, more clinical studies should also be carried out to provide clinical evidence. Furthermore, the current PND prevention and treatment methods related to the gut microbiota are not well developed, so further exploration of these methods is needed.

## Author Contributions

X-QW and HZ were responsible for the study concept, design, and revising the manuscript, and wrote the draft manuscript. HL, X-NL, and C-HY participated in collecting literature. All authors agreed to approved the final manuscript.

## Conflict of Interest

The authors declare that the research was conducted in the absence of any commercial or financial relationships that could be construed as a potential conflict of interest.

## Publisher’s Note

All claims expressed in this article are solely those of the authors and do not necessarily represent those of their affiliated organizations, or those of the publisher, the editors and the reviewers. Any product that may be evaluated in this article, or claim that may be made by its manufacturer, is not guaranteed or endorsed by the publisher.
